# Role perceptions and willingness to engage among older adults in the context of active aging: a qualitative study in Dalian, China

**DOI:** 10.3389/fpubh.2026.1842454

**Published:** 2026-05-29

**Authors:** Xu Lou, Jiajun Leng, Jingyuan Guan, Li Xu, Xiaohua Zhou

**Affiliations:** School of Nursing, Dalian University, Dalian, China

**Keywords:** active aging, population aging, qualitative study, role perception, willingness to engage

## Abstract

**Background:**

Population aging presents profound societal challenges worldwide. Social participation is a core pillar of active aging and a key determinant of health and wellbeing in later life. However, a persistent gap remains between the theoretical recognition of older adults as social resources and their actual level of engagement. This study explored role perceptions, willingness for social engagement, and related influences among urban older adults in China, aiming to inform strategies that promote productive and active aging.

**Methods:**

A descriptive qualitative study, underpinned by a constructivist paradigm, was conducted in Dalian, a super-aged coastal city in China. Between March and June 2025, 22 community-dwelling young-old adults (aged 60–74 years) were recruited through purposive sampling. Data were collected via semi-structured, in-depth interviews and analyzed using Colaizzi’s phenomenological method, with the support of NVivo 14.0 software.

**Results:**

Four interconnected themes emerged, capturing the essence of participants’ experiences: (1) tensions in identity and value—navigating self-perception, societal expectations, and the paradox of satisfaction; (2) heterogeneous readiness for engagement, with preferences rooted in life-course experiences; (3) a multi-level ecosystem of drivers and barriers spanning individual, family, organizational, socio-cultural, and institutional levels; and (4) participant-derived strategies for enabling engagement through coordinated action across government, community, market, and family.

**Conclusion:**

Older adults represent a willing yet heterogeneous social resource. Their engagement potential is constrained by tension between proactive self-perceptions and restrictive societal expectations, alongside multi-level systemic barriers. Translating willingness into sustained action requires targeted, multi-stakeholder interventions that align with their diverse readiness states and life-course-rooted preferences. Such efforts are essential for advancing healthy and active aging.

## 1 Introduction

Population aging has become one of the most significant demographic transformations of the 21st century, exerting far-reaching effects on social, economic, and public health systems worldwide (1). China exemplifies this trend with particular intensity, characterized by both the scale and rapid pace of population aging. By the end of 2024, the population aged 60 years and above in China had reached 310 million, accounting for 22.0% of the total population (2). Liaoning Province, the setting of this study, has entered a “super-aged” stage, with older adults comprising 31.17% of its population (3). Within this province, the coastal city of Dalian faces an even more pronounced challenge, with 31.27% of its registered residents aged 60 years and above (4).

Beyond its demographic scale, China offers a uniquely instructive context for studying older adults’ role perceptions and willingness to engage, for three reasons. First, China is aging “before it has become wealthy” (5), meaning that its social security and older adult care infrastructure remain underdeveloped relative to the speed of demographic change. This places greater pressure on informal family support and community-based initiatives, making the mobilization of older adults’ own capacities a policy imperative rather than merely an aspiration. Second, traditional Confucian norms emphasizing filial piety and family-centered care are being challenged by rapid urbanization, declining household size, and changing intergenerational relationships (6). Although these norms historically positioned older adults as respected older family members, their broader societal roles beyond the family have remained undertheorized and under-supported. Third, China has recently shifted its policy discourse from “providing for older adults” to “making use of older adults”, as reflected in the national active aging strategy (7). Yet the implementation of this shift remains uneven, and the voices of older adults themselves are rarely heard in policy design. Thus, examining how urban older adults in China perceive their potential contributions and what barriers they face can offer insights not only for China but also for other rapidly aging societies undergoing similar cultural and economic transitions.

In response to these demographic shifts, the concept of “active aging” has gained prominence as a guiding international policy framework. The World Health Organization defines active aging as “the process of optimizing opportunities for health, participation, and security to enhance quality of life as people age” (8). This framework represents a paradigm shift away from viewing older adults primarily as care recipients toward recognizing them as active contributors to society. Closely related is the notion of “productive aging,” which emphasizes older adults’ capacity to generate social value through paid or unpaid work, civic engagement, and community participation (9). While active aging broadly encompasses health, participation, and security, productive aging focuses more narrowly on contribution and social utility. The current study centers on the participation pillar of active aging—specifically, older adults’ expectations and willingness regarding social contribution, volunteering, mentoring, and civic engagement—which aligns more closely with the productive aging framework. We therefore use “social engagement” to refer to activities through which older adults contribute their time, skills, and experience to family, community, or society beyond purely leisure or self-care.

A robust body of evidence has linked sustained social participation in later life to favorable health outcomes, including better physical and cognitive function, reduced loneliness, and a lower risk of depression (10, 11). However, despite this growing recognition, a substantial gap persists between the conceptual framing of older adults as social assets or social resources and their actual opportunities for engagement. By “social assets,” we mean the accumulated knowledge, skills, experience, time, and social networks that older adults possess and could potentially deploy for collective benefit. Existing research has predominantly adopted macro-structural or needs-based perspectives, focusing on eldercare services (12, 13), social security systems (14), and integrated care models (15). While these approaches are essential, they offer a relatively limited understanding of older adults as active social agents.

Crucially, research grounded in the subjective perspectives of older adults themselves—examining how they perceive their social roles, how willing they are to engage, and what influences facilitate or constrain their participation—remains underdeveloped. Several gaps are particularly salient. First, regarding role perception, studies have documented that older adults often internalize ageist stereotypes that portray them as dependent and frail (16), but less attention has been paid to how they actively negotiate between self-perceived capability and societal expectations of disengagement. In Confucian-influenced contexts, older adults may experience a unique tension between family-centered role expectations (e.g., grandchild care) and desires for broader societal contribution, yet empirical evidence is limited. Second, regarding willingness and readiness, while surveys have measured general willingness to volunteer (17), qualitative studies that capture the heterogeneity of readiness states—distinguishing those already engaged, those willing but lacking pathways, and those whose willingness is conditional on circumstances—remain scarce. Third, regarding barriers and facilitators, although structural obstacles such as a lack of platforms (9) and age discrimination (18) have been identified, few studies have adopted an ecological perspective that examines how individual, family, organizational, socio-cultural, and institutional influences interact and reinforce each other. The absence of such voice-centered research risks creating a disconnect between policy intentions and older adults’ lived realities, thereby undermining efforts to mobilize this growing “silver human resource” and to realize the documented benefits of social participation.

To address this gap, the present study was conducted in Dalian, a city emblematic of China’s urban aging challenges. Using in-depth qualitative interviews, the study aimed to explore three core questions: (1) How do older adults perceive their roles in an aging society, and what tensions exist between self-perception and perceived societal expectations? (2) What is the nature of their willingness for social engagement, and how does readiness vary across individuals? (3) What influences across multiple levels of their lived experience facilitate or constrain engagement? To guide the interpretation of findings, this study draws on Bronfenbrenner’s Ecological Systems Theory (19) as an overarching framework, attending to role expectations (20), psychological needs (21), and life-course pathways (22) as complementary analytic lenses. These conceptual tools are elaborated in the Methods section. By foregrounding the authentic voices of older adults, this study seeks to provide an empirical foundation for designing more responsive interventions and policies that promote social participation and support the broader goals of active and healthy aging.

## 2 Methods

### 2.1 Study design

A descriptive qualitative study design, underpinned by a constructivist paradigm, was adopted (23). This approach acknowledges that multiple realities are socially constructed through individual experiences and interactions. The study aimed to explore the lived experiences of community-dwelling older adults, focusing on how they perceive their social roles, articulate their willingness to engage socially, and describe the influences that shape this engagement. This phenomenological orientation (24) was chosen because it prioritizes understanding the essence and meaning of a phenomenon from the perspective of those who experience it.

### 2.2 Theoretical framework

This study is guided by Bronfenbrenner’s Ecological Systems Theory (19), which conceptualizes human development as shaped by multiple interacting environmental systems. This framework is particularly suitable for examining older adults’ social engagement because it attends to the nested and interdependent nature of barriers and facilitators at individual, interpersonal, organizational, and cultural levels. Within this overarching ecological perspective, we use three complementary analytic lenses for understanding participants’ experiences that have been highlighted in prior research (20–22): (1) tensions between self-defined and socially prescribed roles (20); (2) basic psychological needs (competence, autonomy, relatedness) that drive engagement (21); and (3) the continuity of accumulated skills and identities over the life course, which shapes heterogeneity in engagement preferences (22). These aspects are not treated as independent theoretical frameworks but as focused analytic lenses nested within the ecological systems model. By integrating them in this way, we aim to provide a coherent, multi-level framework for interpreting how older adults’ willingness to engage is both enabled and constrained.

### 2.3 Participants and sampling

Participants were recruited through purposive sampling from multiple communities in Dalian City, Liaoning Province, China, between March and June 2025. The recruitment sites included communities located in the Zhongshan, Xigang, Shahekou, and Ganjingzi districts, representing the main urban areas of the city. To enhance sample diversity and reflect different living environments of older adults, we selected three types of communities within each district: residential communities, commercial communities, and mixed-use communities. This stratification aimed to capture a broad range of socio-physical contexts.

Eligible participants were recruited through three complementary channels: (1) direct contact in public community spaces such as parks; (2) referrals by community committee staff; and (3) snowball sampling, whereby initial participants recommended other eligible peers. This multi-channel approach was designed to minimize selection bias associated with any single recruitment method. However, we acknowledge that individuals who volunteered to participate might be more socially inclined and have a higher willingness to engage than the average older adult population—a potential recruitment bias that is further addressed in the Limitations section.

Eligibility criteria were as follows: (1) aged 60–74 years (young-old adults); (2) community-dwelling; (3) able to independently perform activities of daily living (ADLs); (4) no significant communication barriers and able to clearly express personal thoughts and experiences; and (5) willing to provide informed consent.

The age range of 60–74 years was intentionally selected to focus on the “young-old” cohort—a population typically transitioning from full-time employment into retirement while retaining relatively good physical and cognitive functioning. To enhance sample heterogeneity, variation was sought in gender, educational attainment, and pre-retirement occupation during recruitment.

Sampling continued until informational saturation was achieved, defined as the point at which no new themes or substantive insights relevant to the research questions emerged. Saturation was reached after three consecutive interviews (participants N20, N21, and N22) yielded no new codes. The final sample comprised 22 participants.

### 2.4 Data collection

A semi-structured interview guide was developed based on the research objectives and a review of relevant literature. Specifically, the guide was informed by key concepts from the productive aging literature (9), role-related concepts (20), the WHO’s active aging framework (8), and stereotype embodiment literature (16). The six domains were designed to operationalize these theoretical constructs as follows: (1) perceptions and societal expectations—drew on ageism literature (16, 18) to explore how participants perceive and internalize societal stereotypes about older adults; (2) self-perceived roles and value—guided by role-related concepts (20) to capture self-defined versus socially prescribed roles; (3) current daily activities—aimed at understanding the actual rhythms of daily life; (4) thoughts and plans on social participation—aligned with the productive aging framework (9) and WHO active aging pillars (8) to elicit willingness and preferred forms of engagement; (5) perceived benefits, difficulties, and concerns—informed by ecological systems theory (19) to identify multi-level barriers and drivers; and (6) additional suggestions—left open-ended to allow participants to raise issues beyond predefined categories, consistent with the exploratory nature of qualitative research.

The guide was designed to be flexible, encouraging participants to share their experiences in their own words. The draft guide was reviewed and refined by two senior researchers with doctoral degrees and more than 10 years of experience in gerontological research. Pilot interviews were then conducted with three older adults who met the inclusion criteria. Feedback from these pilots informed refinements to the wording, sequencing, and probing strategies, resulting in the final interview guide (Supplementary file 1).

Interviews were conducted one-on-one by two trained researchers (XL and JL) in quiet, private settings chosen by participants, most commonly their homes or community offices. Before each interview, the study purpose, procedures, confidentiality assurances, and participants’ right to withdraw were explained, and written informed consent was obtained. Interviews followed a conversational style, allowing flexibility to explore issues raised by participants while maintaining alignment with the guide. Active listening techniques and prompts (e.g., “Could you elaborate on that?”) were used to encourage rich narratives.

Most interviews lasted between 30 and 40 min; two interviews lasted 22 and 28 min, respectively. With participants’ permission, all interviews were audio-recorded. Field notes were taken during and immediately after interviews to document contextual information, non-verbal cues, and initial analytical reflections.

As a conversational strategy to facilitate reflection, participants were invited to rate their overall current life satisfaction on a scale from 1 (very dissatisfied) to 10 (very satisfied). This single-item rating was used solely as a qualitative prompt to initiate a deeper conversation about the sources of their satisfaction, dissatisfaction, and perceived unmet needs. The numerical score itself was not subjected to quantitative analysis; rather, the narratives that followed the rating (e.g., “I give it an 8 because…”) were transcribed and analyzed thematically alongside the broader interview data.

### 2.5 Data analysis

Data analysis followed Colaizzi’s seven-step phenomenological method (24). Audio recordings were transcribed verbatim within 24 h of each interview (XL), with transcript accuracy verified by a second researcher (JG). NVivo 14.0 software was used to support data management.

The seven steps were operationalized as follows: (1) Familiarization—repeated reading of all transcripts to achieve immersion; (2) Identifying significant statements—extraction of statements directly related to role perception, engagement willingness, and related influences; (3) Formulating meanings—each statement was carefully interpreted to derive underlying meanings, striving to remain faithful to participants’ original accounts; (4) Clustering themes—meanings were organized into categories and emergent themes based on conceptual similarity. Discrepancies were resolved through team discussions with senior researchers (XZ and LX); (5) Exhaustive description—all themes were integrated into a comprehensive description of the phenomenon; (6) Fundamental structure—the exhaustive description was distilled to capture the essence of the experience; and (7) Member checking—preliminary findings were reviewed with four participants (N3, N5, N6, N17) to verify that interpretations accurately reflected their experiences. An illustrative example of the analytic process, including significant statements, formulated meanings, and theme development, is provided in Supplementary file 2.

To enhance analytical rigor, independent coding by two researchers (XL and JL) was followed by team discussions to reach consensus. An audit trail documenting analytic decisions was maintained.

Throughout the analysis process, researchers maintained a reflexive stance. A key principle of Colaizzi’s method is fidelity to participants’ accounts, which requires researchers to bracket (set aside) their pre-existing assumptions about aging and social participation. Reflexive diaries were kept to document personal reflections and potential biases, and these were discussed regularly within the research team to ensure interpretations remained grounded in the data rather than imposed by researcher preconceptions.

### 2.6 Rigor and trustworthiness

Trustworthiness was ensured in accordance with the criteria of credibility, dependability, confirmability, and transferability. Credibility was strengthened through prolonged engagement with the data, member checking (as described in step 7 above) to verify interpretations with participants, and peer debriefing within the research team to challenge assumptions and refine themes. Dependability was supported by maintaining a detailed audit trail, including raw data, field notes, coding frameworks, reflexive journal entries, and records of analytical decisions. Confirmability was enhanced through analyst triangulation (independent coding by two researchers) and reflexive discussions to ensure findings emerged from the data rather than from researcher preconceptions. Transferability was facilitated by providing rich contextual descriptions of the study setting (a super-aged city in China), the sampling strategy, participant characteristics (Table 1), and the analytic procedures, allowing readers to assess the applicability of findings to other contexts.

**Table 1:** Participants’ demographic characteristics and current social engagement level (*n* = 22).

ID	Gender	Age (Years)	Education level	Pre-retirement occupation	Current social engagement level^*^
N1	Male	64	Primary school	Sanitation worker	Low
N2	Female	68	College diploma	Primary school teacher	Low
N3	Female	61	College diploma	Doctor	High
N4	Male	63	Primary school	Casual laborer	Medium
N5	Male	63	College diploma	Senior railway engineer	High
N6	Female	62	Junior high school	Community secretary	Medium
N7	Male	74	Bachelor’s degree	Retired government official	Medium
N8	Male	70	Primary school	Enterprise worker	Low
N9	Female	65	Senior high school	Institution employee	Low
N10	Female	74	Junior high school	Self-employed	Low
N11	Female	67	Senior high school	Enterprise worker	Low
N12	Male	66	College diploma	Meat food inspector	Medium
N13	Male	67	Senior high school	Printing factory worker	Low
N14	Female	72	Technical secondary school	Primary school principal	Low
N15	Female	67	Technical secondary school	Librarian	Low
N16	Female	69	Junior high school	Enterprise worker	Low
N17	Male	67	Technical secondary school	Technical school teacher	Medium
N18	Male	66	Technical secondary school	Technical school teacher	High
N19	Male	65	Bachelor’s degree	University teacher	High
N20	Female	70	Technical secondary school	Clinical nurse/university teacher	Medium
N21	Male	68	Postgraduate	University teacher	High
N22	Male	72	Senior high school	Accountant	Low

^*^Level assessed based on participant self-description: Low = primarily family duties and personal leisure; Medium = occasional, informal community involvement or volunteering; High = regular, structured social contribution (re-employment, organized volunteering).

### 2.7 Ethical considerations

This study was conducted in accordance with the Declaration of Helsinki and filed with the Academic Committee of the School of Nursing, Dalian University (record No. HL-2025007). Written informed consent was obtained from all participants. Data were anonymized, and confidentiality was strictly maintained.

## 3 Results

### 3.1 Participant characteristics and analytic overview

Twenty-two community-dwelling older adults participated in the study, including 12 males and 10 females, with ages ranging from 61 to 74 years. Participants represented diverse educational backgrounds, pre-retirement occupations, and current living situations (Table 1).

Following Colaizzi’s phenomenological method, analysis revealed four interconnected themes that capture the essence of older adults’ experiences regarding social role perceptions and engagement willingness: (1) tensions in identity and value*—*navigating self-perception, societal expectations, and the paradox of satisfaction; (2) heterogeneous readiness and preferred forms of engagement; (3) a multi-level ecosystem of drivers and barriers; and (4) participant-derived strategies for enabling engagement.

### 3.2 Theme 1: tensions in identity and value

This theme captures the fundamental tensions participants experienced between their internal sense of continued capability, perceived societal expectations, and the resulting paradox of feeling satisfied yet unfulfilled.

#### 3.2.1 Self-perception: the capable self and the fading self

Participants described multiple, often coexisting self-perceptions. A strong sense of continued capability and desire to contribute was common, especially among those with professional backgrounds. A retired engineer stated: “*We still have experience and insight; we can continue to create value. My mind is as sharp as it was 20 years ago, even if my back isn’t*.” (N5) A retired physician echoed: “*Many of us still have abundant energy and a strong desire to contribute to society. It’s not about money; it’s about feeling that what you know still matters*.” (N3) These statements reveal that participants not only recognized their own competencies but also actively sought meaningful outlets for their skills.

However, this proactive self-view often coexisted with anxiety about decline and marginalization. A former casual laborer expressed: “*I can’t handle heavy work anymore. I look in the mirror and see someone who is still strong inside, but the world outside sees someone who is weak. Society seems to be phasing us out*.” (N4) For some, these contrasting perceptions created unresolved internal tension: “*I feel useful, but I’m not sure anyone else does*.” (N16). This contrast highlights that participants’ sense of agency was not solely an internal matter; it was constantly mediated by perceived external judgments.

#### 3.2.2 Perceived societal expectations: the script of disengagement

Participants were acutely aware of what they believed society expected of them. A retired government official described this script with clarity: “*The common view is that older adults should focus on family, take care of grandchildren, manage their health, and avoid too much social involvement so as not to burden others. Society tells us: ‘You’ve done your part, now step aside*.’” (N7) The power of this script lay in its subtle, internalized effect. A retired teacher reflected: “*Sometimes I think about doing more, like teaching or volunteering. But then I wonder—am I being unrealistic? Shouldn’t I just be grateful to be healthy and leave things to the younger generation?*” (N2) This internal questioning demonstrates how societal expectations can be absorbed into one’s own self-evaluation, constraining aspirations even before any external barrier is encountered.

#### 3.2.3 Aspirational role constructions despite constraints

Despite these constraints, participants articulated proactive visions of roles they could play. A former community secretary envisioned mutual support: “*We could organize neighborhood mutual aid—us ‘younger’ older people helping the older ones. Shopping, chatting, just checking in. I could set up interest groups, organize collective activities for the neighbors*.” (N6) A retired university teacher imagined contributing to public discourse: “*We have decades of experience. Why shouldn’t that be used? We could serve on advisory committees, help shape policy. Our voices matter*.” (N21) These statements show that participants did not simply accept the disengagement script; they actively imagined alternative contributions, suggesting that latent willingness is widespread but awaits structural support.

#### 3.2.4 The paradox of satisfaction: contentment with daily life yet yearning for purpose

When reflecting on their overall life satisfaction, participants typically affirmed what they had—financial security, health, and family harmony. A retired woman spoke of peaceful contentment: “*I’m busy, but it’s a peaceful busy*.” (N10) A man caring for his wife described a routine filled with family duties: “*Every day revolves around my wife and the home… When the kids need help—picking up a grandchild, bringing over food—I do that too*.” (N13) Yet as conversations deepened, a more complex picture emerged. A retired teacher expressed poignantly: “*My knowledge and experience are just left idle. Sometimes I feel a lack of being needed. It’s not that I’m unhappy—I have everything I need. But something is missing*.” (N2) A retired engineer described his skills as a resource going to waste: “*I spent forty years solving problems. Now the only problems I solve are whether to have rice or noodles for dinner. It feels… small. I miss feeling useful in a bigger way*.” (N5).

These narratives reveal a profound insight: satisfaction with basic security did not equate to fulfillment of purpose. Participants could be genuinely content with their material and relational circumstances while simultaneously yearning for meaningful contribution. This paradox lies at the heart of their experience and explains why many remained willing to engage despite reporting high life satisfaction.

### 3.3 Theme 2: heterogeneous readiness and preferred forms of engagement

All participants expressed some openness to engagement, but this varied markedly in degree and form.

#### 3.3.1 Three readiness states

We identified three distinct readiness states: (1) Proactively engaged—participants who had already found ways to contribute. A retired university teacher described: “*I teach English at the community library and participate in community advisory meetings. Teaching was my life for forty years; why would I stop? It’s about continuing to be who I am*.” (N21) (2) Positively willing—a substantial number of participants fell into this category; they were genuinely interested but lacked pathways. A retired physician explained: “*I’m very willing. I could offer health consultations, help people understand their medical examination reports. But I don’t know how to start. The desire is there, but the door isn’t*.” (N3) (3) Conditionally willing—genuine desire constrained by circumstances (e.g., caregiving, health). A man caring for his wife noted: “*My heart is willing. But I have to care for my wife first. If her health improves, or if the community has something flexible, I’d be happy to join*.” (N13) These categories highlight that willingness alone is insufficient; practical pathways and flexible arrangements are essential to translate intention into action.

#### 3.3.2 Preferred forms of engagement

Preferences clustered into four archetypes, each rooted in life experience: (1) Knowledge and skills transmission*—*preferred by those with professional backgrounds. A former food safety inspector envisioned: “*What I’d really love is community food safety education. Public lectures, simple materials teaching people how to check food quality. I have this knowledge; why shouldn’t I share it?*” (N12) (2) Volunteering and practical tasks*—*attracted those preferring hands-on contributions. A former factory worker said simply: “*I’m not a teacher, but I have two hands and I’m willing to use them*.” (N8) (3) Intellectual and advisory roles*—*preferred by a smaller, highly educated group. A retired government official proposed: “*A ‘silver think tank’—that’s what we need. Formal channels for our voices in policy*.” (N7) (4) Organizational and coordination roles*—*appealed to those with experience mobilizing people. A former community secretary enthused: “*I want to get neighborhood mutual aid going! I know how to organize, how to connect people*.” (N6).

These archetypes demonstrate that heterogeneity is not random but systematically linked to participants’ prior occupational and social roles. Interventions should therefore map individual skills rather than offer generic opportunities. Comparing across occupational groups, participants with higher education or professional credentials (e.g., teachers, engineers, physicians, university faculty) consistently expressed preferences for knowledge transmission or advisory roles. In contrast, those with manual or service backgrounds (e.g., factory workers, sanitation workers, self-employed individuals) preferred practical volunteering or organizational coordination. This pattern suggests that life-course accumulated skills and social capital—rather than age alone—are key determinants of engagement preferences.

### 3.4 Theme 3: a multi-level ecosystem of drivers and barriers

Participants’ willingness was shaped by an interconnected system of forces across multiple levels of their lives.

#### 3.4.1 Intrinsic drivers

Three intrinsic drivers were prominent: (1) Self-actualization and social value*—*A retired librarian explained: “*What matters most is feeling that I’m still useful, that someone still needs me. It’s about knowing that if I weren’t here, someone would notice*.” (N15) (2) Social connection*—*A widowed participant described her isolation: “*If I had something to do, somewhere to go, people to see… it would make a difference. Being alive, but there’s no meaning to it*.” (N16) (3) Health maintenance*—*A retired physician observed: “*Staying socially active keeps your brain engaged. People who have purpose age better*.” (N3) These drivers are not mutually exclusive; often they reinforce each other. For example, feeling useful also provides social connection, and both contribute to perceived health benefits.

#### 3.4.2 Barriers across ecological levels

Barriers operated at multiple, interconnected levels: (1) Individual level—limited energy and fear of causing trouble. A 72-year-old explained: “*My body and energy—they’re the main limitation. I have to think: can I physically manage it?*” (N22) (2) Family level—caregiving responsibilities. A grandmother caring for her granddaughter noted: “*There’s no space for anything else. I’m not complaining, but there’s no room for me to do anything else*.” (N14) While caregiving for grandchildren can provide older adults with a sense of meaning, generativity, and social connection in other cultural contexts, participants in this study predominantly framed these responsibilities as time-consuming obligations that left little room for external social participation. This may reflect the intensive nature of grandchild care in urban China, where many older adults serve as primary caregivers due to parental work commitments. (3) Organizational level—absence of platforms. A retired university teacher expressed frustration: “*There’s no efficient platform to match skills with needs. I have expertise, time, willingness. But where do I go?*” (N19) (4) Socio-cultural level—ageism. A retired government official observed: “*There’s a subtle message that we’re past our prime. After a while, you start to wonder if maybe they’re right*.” (N7) (5) Institutional level—policy gaps and inadequate safeguards. A former community secretary noted: “*We need protection. Without clear rules, without insurance, people hesitate*.” (N6).

Crucially, these barriers were mutually reinforcing. Individual hesitation was amplified by family obligations, reinforced by organizational gaps, legitimated by cultural assumptions, and encoded in institutional policies. Participants were blocked not by any single barrier but by a whole system of constraints. This systemic quality explains why isolated interventions (e.g., simply creating a few volunteer positions) are unlikely to succeed.

### 3.5 Theme 4: participant-derived strategies for enabling engagement

Despite these barriers, participants articulated a coherent vision of how engagement could be enabled, assigning roles to different actors.

#### 3.5.1 Government: institutional design and safeguards

Participants called for formal participation channels and protection. A retired official proposed: “*Establish a ‘silver think tank’—formal, regular channels for our voices in policy. And provide insurance—remove the fear that something going wrong will leave us vulnerable*.” (N7).

#### 3.5.2 Community: platforms and support

Community was seen as the most important arena. A retired teacher envisioned: “*Build a database of retired professionals—their skills, interests, availability. Match people with opportunities. And give us a place, with someone whose job is to organize things*.” (N19) Another participant emphasized flexibility: “*Activities need to accommodate those with family responsibilities or health limitations. One-time projects, drop-in opportunities—things we can do when we can*.” (N14).

#### 3.5.3 Market and media: reshaping recognition

Businesses could create opportunities, while media could reshape perceptions. A retired self-employed individual said: “*Companies should hire us! Not for full-time pressure, but advisory roles, mentoring. And the media should show us differently—not just frailty, but contribution, vitality*.” (N10).

#### 3.5.4 Family: emotional support and technological bridging

Finally, participants looked to families as enablers. A retired nurse hoped for encouragement: “*I wish my children would support me. Say ‘Mom, that’s great, we’re proud of you.’ Don’t see my activities as taking away from the family—see them as adding to who I am*.” (N20) A retired librarian called for patience with technology: “*Young people—be patient. Teach us how to use the new tools. Like we taught you, when you were young*.” (N15).

In summary, participants were not passive victims of barriers; they actively envisioned a multi-actor framework that could transform their willingness into sustained engagement. This vision, emerging from their lived experience, offers useful directions for policy and practice.

## 4 Discussion

This qualitative study examined role perceptions, willingness for social engagement, and related influences among community-dwelling older adults in Dalian, China. Guided by Bronfenbrenner’s Ecological Systems Theory (19), we interpret the findings as reflecting interactions across multiple levels of participants’ lived environments. Four interconnected themes emerged: tensions in identity and value; heterogeneous readiness and preferred forms of engagement; a multi-level ecosystem of drivers and barriers; and participant-derived strategies for enabling engagement. Below, we discuss these findings within the proposed ecological framework.

### 4.1 Microsystem: individual and family-level dynamics—role tensions and the satisfaction paradox

At the microsystem level (immediate environment), participants experienced a pronounced tension between their intrinsic sense of continued capability and perceived societal expectations of disengagement. This reflects a conflict between self-defined roles (e.g., contributor, advisor) and externally imposed role expectations (e.g., family-centered retiree) (20). The perceived script functions as a form of internalized ageism (25)—a quiet assumption that constrains the legitimacy of aspirations for continued contribution. Several participants described how they questioned their own ambitions, wondering whether wanting to do more was unrealistic or whether they should simply leave things to the younger generation. This internal questioning demonstrates how societal expectations can be absorbed into self-evaluation, limiting agency even before external barriers are encountered.

Simultaneously, at this micro level, participants expressed genuine satisfaction grounded in security and family harmony, yet many carried a sense that something essential was missing. This paradox can be understood by considering basic psychological needs (competence, autonomy, relatedness); their satisfaction leads to wellbeing (21). Satisfaction with family harmony reflects the need for relatedness, whereas the yearning for purpose reflects the salience of competence and autonomy needs. Once basic needs are met, these higher-order needs become more salient. This finding suggests that older adults do not seek activity per se, but rather engagement that affirms personal value (26). The feeling of “not being needed” indicates a gap between subjective capability and available roles—a gap that conventional satisfaction measures cannot capture. This yearning reflects a persistent need to guide and contribute to future generations (27), which aligns with the competence and autonomy needs described above.

### 4.2 Mesosystem and exosystem: interactions between family, community, and organizational platforms

The mesosystem (interactions between microsystems) and exosystem (settings that indirectly affect the individual) were particularly salient in participants’ accounts of heterogeneous readiness and barriers. The identification of three readiness states—proactively engaged, positively willing, and conditionally willing—alongside four engagement archetypes (knowledge transmission, practical volunteering, advisory roles, and organizational coordination) underscores heterogeneity. Preferences can be understood as extensions of accumulated skills and identities over the life course (22) (micro-level), yet these individual predispositions intersected with organizational gaps (exo-level). Participants with professional backgrounds tended to favor advisory or knowledge-sharing roles, while those with experience in organizing people preferred coordination roles. This aligns with evidence that occupational trajectories shape post-retirement civic participation (28).

However, these individual preferences intersected with organizational gaps. Participants across all readiness states emphasized the absence of intermediary platforms (exosystem) that could match their skills with community needs. The proposed “silver talent database” represents a participant-derived solution for bridging this gap. Such alignment between personal capital and engagement roles has been associated with higher-quality participation and greater psychological benefits (29). Notably, family obligations (microsystem) intersected with organizational gaps (exosystem) to produce compounded constraints: the lack of flexible opportunities that could accommodate caregiving responsibilities left many conditionally willing participants stuck despite genuine desire. This meso-exo interaction highlights the need for interventions that address both individual circumstances and structural opportunities. It should be noted, however, that family caregiving is not universally restrictive. Prior research has shown that grandchild care can enhance older adults’ sense of purpose and generativity (27). The predominance of the barrier framing in our data likely reflects the specific intensity of caregiving in urban Chinese families, where older adults are often the sole caregivers for extended hours daily, leaving little discretionary time. Interventions that provide respite care or shared caregiving arrangements could help transform family roles from a constraint into a source of dual contribution.

### 4.3 Macrosystem: cultural ageism and the societal script of disengagement

At the macrosystem level (cultural values, beliefs, and ideologies), participants confronted ageist assumptions that delegitimized their aspirations for contribution. While ageist stereotypes are documented cross-culturally (18), the content of expectations identified here appears shaped by a distinct cultural configuration. Traditional Confucian norms emphasizing family responsibility intersect with productivity-oriented ageism, creating a dual expectation: older adults are expected to prioritize family care while simultaneously being viewed as lacking broader social utility. This cultural script was internalized by many participants, who reported feeling that society saw them as “past their prime” or that they should “step aside” for younger generations. This differs from contexts where civic roles for older adults have received sustained institutional recognition (9, 30). Promoting active aging in urban China therefore requires not only expanding opportunities but reframing culturally sanctioned definitions of “contribution” to encompass community and societal engagement beyond the family. Media campaigns that portray older adults as active contributors rather than passive care recipients could gradually shift this macrosystem narrative.

### 4.4 Interconnections across levels: toward an integrated ecological intervention model

Crucially, the barriers participants described were mutually reinforcing across levels. Individual hesitation (micro) was amplified by family obligations (micro), reinforced by organizational gaps (exo), legitimated by cultural assumptions (macro), and encoded in institutional policies (macro). Participants were blocked not by any single barrier but by a whole system of constraints. Strategies targeting only one level—for example, creating volunteer programs without addressing cultural ageism or providing insurance protection—are unlikely to succeed.

A notable contribution of this study is that participants themselves articulated a coherent, multi-actor framework for addressing these barriers: government (institutional design and safeguards), community (platform building and flexible programming), market and media (opportunity creation and cultural change), and family (emotional support and technological bridging). This participant-derived framework lends strong emic validity to an ecological intervention approach, suggesting that effective policies may need to intervene simultaneously across micro, meso, exo, and macrosystems.

### 4.5 Summary of contributions

This study makes three specific contributions to the literature on older adults’ social engagement. First, it advances the conceptual understanding of the “willingness-action” gap by empirically distinguishing three readiness states (proactively engaged, positively willing, conditionally willing) and revealing a readiness paradox: high life satisfaction can coexist with an unspoken yearning for purpose. Second, it provides an empirically grounded typology of four engagement archetypes rooted in life-course experiences (knowledge transmission, practical volunteering, advisory roles, and organizational coordination), moving beyond generic “volunteering” categories and demonstrating how prior occupational and social roles shape preferred forms of contribution. Third, it applies Bronfenbrenner’s ecological systems theory to show, from older adults’ own perspectives, how barriers across individual, family, organizational, socio-cultural, and institutional levels mutually reinforce each other—and how participants themselves envision a multi-stakeholder solution.

### 4.6 Implications for practice, research, and policy

For practice, community organizations should adopt strengths-based engagement—mapping older adults’ skills, readiness states, and preferences. Programs must be flexible, accommodating those with family responsibilities or health limitations through drop-in opportunities and one-time projects.

For research, future studies should quantify the prevalence of identified archetypes and readiness states in larger samples. Longitudinal designs could examine how engagement influences health outcomes over time and how readiness evolves. Comparative studies across cultural contexts could illuminate how the macrosystem script of disengagement varies and might be reshaped.

For policy, priorities include: removing age-restrictive regulations; establishing risk protection (e.g., accident insurance) for older volunteers; investing in municipal engagement platforms; and supporting public narratives recognizing diverse forms of contribution. Policy should incentivize intergenerational collaboration, leveraging complementary strengths of both generations.

## 5 Conclusion

This study demonstrates that older adults in urban China are a willing yet heterogeneous social resource, with their engagement potential constrained by tensions between proactive self-perceptions and restrictive societal expectations, as well as multi-level systemic barriers. The coexistence of quiet contentment with unspoken yearning reveals that satisfaction with basic security does not equate to fulfillment of basic psychological needs (competence, autonomy, relatedness). Effective promotion of active aging requires targeted empowerment—aligned with diverse capacities, readiness states, and preferences rooted in life-course experiences—supported by an integrated, multi-stakeholder response across family, community, government, market, media, and cultural environments.

## 6 Limitations

Several limitations should be acknowledged. First, participants were recruited exclusively from urban communities in Dalian, a super-aged city with specific socio-economic and cultural characteristics. This may limit the transferability of findings to rural settings or regions with different demographic profiles. Second, the study focused only on the young-old age group (60–74 years). Older adults aged 75 years and above may experience agency decline, disengagement, and frailty differently, so our findings may not apply to them. Third, as a qualitative study relying on self-reported data, our findings may be subject to social desirability and self-selection biases (individuals who volunteered might have been more socially inclined and willing to engage), despite efforts to minimize these through rapport-building and confidentiality. Fourth, the study design allows for rich description but does not permit quantification of theme prevalence or causal inference. Fifth, the study primarily reflects the demand-side perspective of older adults themselves; future research incorporating supply-side stakeholders—community administrators, policymakers, employers—is needed to provide a more comprehensive understanding of engagement systems.

## Data Availability

The datasets presented in this article are not readily available because the qualitative interview data generated in this study are not publicly available due to ethical constraints protecting participant privacy and confidentiality. Data will be made available by the corresponding author upon reasonable request, without undue reservation. All other cited data sources are referenced in the manuscript. Requests to access the datasets should be directed to XZ, zhouxiaohua@dlu.edu.cn.
