# Correction: Role perceptions and willingness to engage among older adults in the context of active aging: a qualitative study in Dalian, China

**DOI:** 10.3389/fpubh.2026.1945637

**Published:** 2026-08-05

**Authors:** Xu Lou, Jiajun Leng, Jingyuan Guan, Li Xu, Xiaohua Zhou

**Affiliations:** School of Nursing, Dalian University, Dalian, China

**Keywords:** active aging, population aging, qualitative study, role perception, willingness to engage

The name of the committee was erroneously given as “the Ethics Review Committee of Dalian University”. It should be “the Academic Committee of the School of Nursing, Dalian University”.

In section 2.7 Ethical considerations, the sentence was:

“This study was conducted in accordance with the Declaration of Helsinki. Ethical approval was obtained from the Ethics Review Committee of Dalian University (Approval No. HL-2025007).”

This has been corrected to:

“This study was conducted in accordance with the Declaration of Helsinki and filed with the Academic Committee of the School of Nursing, Dalian University (record No. HL-2025007).”

The Ethics statement previously read:

“The studies involving humans were approved by Ethics Review Committee of Dalian University (Approval No. HL-2025007). The studies were conducted in accordance with the local legislation and institutional requirements. The participants provided their written informed consent to participate in this study.”

The corrected Ethics statement is:

“This study involved only qualitative interviews and did not involve biological sample collection, drug intervention, or invasive procedures, with a risk level not exceeding minimal risk. It was filed with the Academic Committee of the School of Nursing, Dalian University (record No. HL-2025007). The studies were conducted in accordance with the local legislation and institutional requirements. The participants provided their written informed consent to participate in this study.”

The original version of this article has been updated.

