# Supplementary material for: Role perceptions and willingness to engage among older adults in the context of active aging: a qualitative study in Dalian, China
Source: Front Public Health. 2026 May 29;14:1842454. doi: 10.3389/fpubh.2026.1842454 (PMC13259976; doi:10.3389/fpubh.2026.1842454)
Supplement: Supplementary file 1 [file Supplementary_File1.DOCX]

**A semi-structured interview guide for the study “Role Perceptions and Willingness to Engage among Older Adults in the Context of Active Aging: A Qualitative Study in Dalian, China”**

**1.** Perceptions and Social Expectations:

- What comes to mind first when you hear the term “older adult”?
- What do you think society generally believes older adults should do?

**2.** Self-perceived Roles and Value:

- In your view, what roles and value can older adults like yourself play in the family, community, and society?

**3.** Current Daily Life and Self-assessment:

- What are your main daily activities currently?
- If you were to rate your current life on a scale from 1 to 10, what score would you give and why?

**4.** Thoughts and Plans on Social Participation:

- Beyond your current life, what are your thoughts or plans regarding social participation (e.g., contributing to the community or others)?
- If possible, what type of participation would you most like to try?

**5.** Perceived Benefits, Difficulties, and Concerns:

- What benefits do you think participating in these activities would bring you?
- What are the main difficulties or concerns you have about participating more?

**6.** Additional Suggestions for “Productive Aging”:

- Regarding how to better enable older adults to realize their value and achieve “productive aging”, do you have any other opinions or suggestions?
