# Supplementary material for: Role perceptions and willingness to engage among older adults in the context of active aging: a qualitative study in Dalian, China
Source: Front Public Health. 2026 May 29;14:1842454. doi: 10.3389/fpubh.2026.1842454 (PMC13259976; doi:10.3389/fpubh.2026.1842454)
Supplement: Supplementary file 2 [file Supplementary_File2.DOCX]

**Supplementary File 2: Illustrative Example of the Analytic Process**

| **Significant Statement (Participant ID)** | **Formulated Meaning (Code)** | **Sub-category / Category** | **Overarching Theme** |
| --- | --- | --- | --- |
| “*We still have experience and insight; we can continue to create value. My mind is as sharp as it was 20 years ago, even if my back isn’t.*” (N5) | Desire for continued contribution; sense of capability despite physical decline | Self-Perception as Contributor | Theme 1: The Duality of Role Perception |
| “*There is no efficient platform to match the skills of older adults with societal needs.*” (N19) | Perception of a structural gap between personal capacity and societal opportunity. | Organizational Barrier: “Lack of Platforms” | Theme 4: A Multi-Level Ecosystem of Drivers and Barriers |
| “*We could initiate ‘neighborhood mutual support for aging*’.” (N6) | Proactive idea to create community-based, peer-led support systems. | Proposed Solution: Community as Organizer | Theme 5: Participant-Derived Strategies for Enabling Engagement |

This table provides an example of how significant statements were interpreted and clustered into themes following Colaizzi’s phenomenological method. It is intended to illustrate the analytic process, not to present exhaustive coding.
